# Cervical Characterization with Tactile-Ultrasound Probe

**DOI:** 10.4236/ojog.2020.101008

**Published:** 2020-01-08

**Authors:** Vladimir Egorov, Todd Rosen, Heather van Raalte, Viktors Kurtenoks

**Affiliations:** 1 Advanced Tactile Imaging, Trenton, NJ, USA; 2 Department of Obstetrics, Rutgers Robert Wood Johnson Medical School, New Brunswick, NJ, USA; 3 Princeton Urogynecology, Princeton, NJ, USA

**Keywords:** Cervical Elasticity, Cervical Length, Tactile, Ultrasound, Elastography, Premature Cervical Softening, Spontaneous Preterm Delivery

## Abstract

**Background:**

Premature cervical softening and shortening may be considered an early mechanical failure that predisposes to preterm birth. Preliminary clinical studies demonstrate that cervical elastography may be able to quantify this phenomenon and predict spontaneous preterm delivery.

**Objective:**

To explore a new approach for cervix elasticity and length measurements with tactile-ultrasound probe.

**Methods:**

Cervix probe has tactile array and ultrasound transducer designed to apply controllable load to cervix and acquire stress-strain data for calculation of cervical elasticity (Young’s modulus) and cervical length for four cervix sectors. Average values, standard deviations, intraclass correlation coefficients and the 95% limits of agreement (Bland-Altman plots) were estimated.

**Results:**

Ten non-pregnant and ten pregnant women were examined with the probe. The study with non-pregnant women demonstrated a reliable acquisition of the tactile signals. The ultrasound signals had a prolonged appearance; identification of the internal os of the cervix in these signals was not reliable. The study with pregnant women with the gestational age of 25.4 ± 2.3 weeks demonstrated reliable data acquisition with real-time visualization of the ultrasound signals. Average values for cervical elasticity and standard deviations of 19.7 ± 15.4 kPa and length of 30.7 ± 6.6 mm were calculated based on two measurements per 4 sectors. Measurement repeatability calculated as intraclass correlation coefficients between two measurements at the same cervix sector on pregnant women was found to be 0.97 for cervical elasticity and 0.93 for the cervical length. The 95% limits of agreement of 1) cervical elasticity were from −22.4% to +14.9%, and 2) cervical length from −13.3% to +16.5%.

**Conclusions:**

This study demonstrated clinically acceptable measurement performance and reproducibility. The availability of stress-strain data allowed the computation of cervical elasticity and length. This approach has the potential to provide cervical markers to predict spontaneous preterm delivery.

## 1. Introduction

Preterm delivery is a leading cause of infant mortality and morbidity. It is estimated that annually about 15 million infants are born preterm worldwide [1]. Across 184 countries, the rate of preterm birth ranges from 5% to 18%, with almost 1 million children dying each year due to complications in preterm birth. Of the 14 million survivors per year, many face a lifetime of disability, including learning disorders, as well as visual and hearing impairments [2]. Long-term complications include cognitive disorders, behavioral problems, and cerebral palsy [3] [4] [5] [6]. These consequences imply devastating financial, social, and emotional effects on the parents and/or the affected children. In the US, the short-term hospital costs during the first year of life of preterm birth/low-birth-weight infants were estimated to be at $5.8 billion and the estimated annual societal economic burden in the US is, at a minimum, $26.2 billion [7]. Identifying women at risk for spontaneous preterm delivery (SPTD) remains an issue of paramount importance [8] [9] [10].

Premature cervical softening and shortening may be considered an early mechanical failure that predisposes to preterm birth [11]. The digital cervical score [12] and Bishop score [13] as predictors of SPTD have demonstrated low diagnostic accuracy (61% - 68%) [14]. Even though numerous risk factors associated with SPTD have been identified in previous work, the ability to accurately predict when labor will occur remains elusive [15] [16] [17] [18]. In a well-regarded, large observational cohort study, serial transvaginal ultrasound cervical length and quantitative vaginal fetal fibronectin had low predictive accuracy for SPTD among nulliparous women [19]. Recent clinical findings suggest that cervical elastography may be a more useful test to predict preterm delivery [20]–[25]. Cervical elasticity may better assess microstructural changes in the cervix that predict preterm birth [26], and therefore, using cervical stiffness and length as part of a multiple marker screen to predict SPTD has the potential to improve on current methods.

The objective of this study was to assess a new approach for cervical elasticity and length measurements based on the acquisition of stress-strain data by a cervix probe with tactile and ultrasound transducers in a clinical study.

The pilot study was conducted with the use of a new cervical probe.

## 2. Material and Methods

### 2.1. Study Design

Between July 2017 and February 2018, 10 non-pregnant women and 10 pregnant women at 22 – 29 weeks of pregnancy were enrolled into a pilot clinical study and examined with the Cervix Monitor (CM). Written informed consent was obtained for this Institutional Review Board approved observational study (clinical trials identifiers ). The study objectives were: 1) to assess the device performance, 2) to assess the potential risks of the CM to pregnant women and fetuses, first with non-pregnant women followed by pregnant subjects, and 3) to verify the proposed data collection and examination techniques. The study with pregnant women followed the assessment of the risks of the CM examination procedure with non-pregnant women, as required by the Code of Federal Regulations, Title 45, §46.204(a). The inclusion criteria were that the participants had to be adult women, aged between 21 – 44 years, who were not pregnant for the first phase of the study, and pregnant in the second phase. Exclusion criteria included the presence of active cancer of the colon, rectum wall, cervix, vagina, uterus or bladder; ongoing or prior radiation therapy for abdominal or pelvic cancer; recent (less than 12 months) pelvic surgery; surgically absent uterus, rectum or bladder; significant circulatory or cardiac conditions that could cause excessive risk from the examination as determined by the attending physician; severe abdominal or pelvic adhesions preventing access to pertinent anatomy; known or suspected bleeding disorders; HIV or hepatitis B positive serology; warty lesions on the vulva; extensive varicose veins on the vulva; active skin infection or ulceration within the vagina/vulva (Herpes infection); and the presence of a vaginal septum. In addition, pregnant women deemed to be at a high-risk owing to a maternal or fetal condition were excluded. The participants’ age, height, weight, gestational age and parity distribution data were collected. The total study workflow comprised of the following steps: 1) Recruiting women who routinely undergo gynecological or obstetric examination; 2) Acquisition of clinical information related to the studied cases by standard clinical means; 3) Performing a CM examination in a lithotomy position; and 4) Analyzing the CM data. All the participating women were asked to complete a questionnaire about their pain and comfort levels during the CM examination.

### 2.2. Cervix Monitor (CM)

The Vaginal Tactile Imager (VTI) was initially developed as a biomechanical mapping device to assess vaginal and pelvic floor conditions [27] [28]. It allows the acquisition of cervical pressure response signals but does not allow cervical elasticity and length measurements. The CM has a drastically revised design in most of the engineering and clinical aspects.

**Figure 1.**
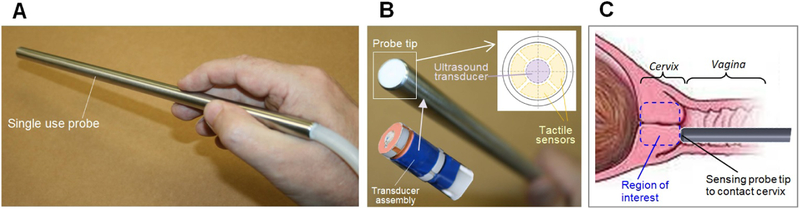
Cervical probe. (A) General view of the Cervix Monitor probe; (B) Probe tip with tactile and ultrasound transducers; (C) Probe positioning at cervical measurement.

The CM was designed as a cart-based device with a medical grade touchscreen computer (Tangent, CA) and a detachable single-use cervix probe. The CM probe contains a tactile array with four sensors and an ultrasound transducer as shown in Figure 1. The ultrasound 3.0 MHz transducer, working in the pulse-echo mode with data acquisition resolution of 20 ns (50 MHz sample rate), measures 3.5 mm in diameter. Biocompatible, two-component silicones (NuSil Technology, CA) were employed to provide sensor assembly functionality, durability, stability and mechanical protection. A proprietary printed circuit board was designed to perform the dual functions of tactile signal acquisition and generation/acquisition of synchronized ultrasound signals. Its key features are to serve four tactile/pressure sensors and one ultrasound transducer at 100 data frames per second. Figure 1 presents the CM probe used in this study.

The pressure measurement noise level was below 25 Pa within the operational range of 40 kPa. The ultrasound transmitting pulses had a peak amplitude below 50 V, a length less than 1 μs, which provide acoustic power significantly below the limits established by the FDA for ultrasound emission in obstetrics: spatial-peak temporal-average Ispta = 94 (mW/cm^2^), spatial-peak pulse-average intensity Isppa = 190 (W/cm^2^), and mechanical index MI = 1.9. Medical grade 316 stainless steel, used in the production of surgical instruments, was used to fabricate the probe body (Figure 1). The CM software interface allows real-time observation of the cervical ultrasound signal as well as applied pressure. The ultrasound peak position for cervix internal os signal was calculated with the use of a signal envelope after the Gaussian complex wavelet filtering [29] at 3 MHz frequency. The cervical elasticity was calculated as a ratio of the applied load (stress) to the surface of the cervix from the CM probe to the resultant changes of the cervical length (strain). This approach was validated with the soft tissue models in bench testing and verification. Young’s modulus was calculated from the stress-strain data based on a semi-infinitive linear elastic model [30] [31].

### 2.3. Examination Procedure

The CM examination procedures followed the following steps: 1) Inserting the speculum into the vagina to provide appropriate visualization and access to the cervix; 2) Performing double CM measurements at 3, 6, 9, and 12 o’clock, specifying the probe tip location on cervix on the CM touchscreen display; 3) Reviewing of the measurement results (ultrasound reflected waves and applied loads); and 4) Removal of the probe and speculum from the vagina.

### 2.4. Statistical Analysis

Measurement repeatability between two measurements at the same cervix sector on pregnant women was assessed with an intraclass correlation coefficient (ICC), as the correlation between any two measurements made on the same subject [32]. The following parameters were calculated as described by Bland and Altman [33]: 1) Bias (*i.e*., the mean of the proportionate difference [the difference between two CM measurements divided by the average value of two measurements]); 2) Precision (*i.e*., the standard deviation of the difference between the two measurements); and 3) Proportionate 95% limits of agreement (*i.e*., 1.96 times the standard deviation of the mean of the proportionate difference). The average values and standard deviations were calculated. Statistical analysis was performed with MATLAB version R2018a (MathWorks, MA).

## 3. Results

### 3.1. Study with Non-Pregnant Women

The study with non-pregnant women demonstrated reliable acquisition of the tactile signals. However, the ultrasound reflected signals had prolonged appearance; identification of the cervix internal os in these signals was not reliable. However, the signal post-processing of 8 of the 10 cases allowed the calculation of average cervical elasticity of 54 ± 17 kPa and length of 42 ± 13 mm. In 2 of the 10 cases, we found very low returned ultrasound signal amplitudes which offered no possibility for the elasticity assessment. These were expected difficulties with CM signal acquisition due to the cervical anatomy and positioning in non-pregnant women. The average pain level for 10 cases was 1.1 on the scale from 1 to 4 (1: none, 2: mildly painful, 3: painful, 4: severely painful). The comfort level was adjudged at 2.2 on the scale from 1 to 3, *i.e*. essentially similar to manual palpation (1: more comfortable than manual palpation, 2: the same, 3: less comfortable).

### 3.2. Study with Pregnant Women

**Figure 2.**
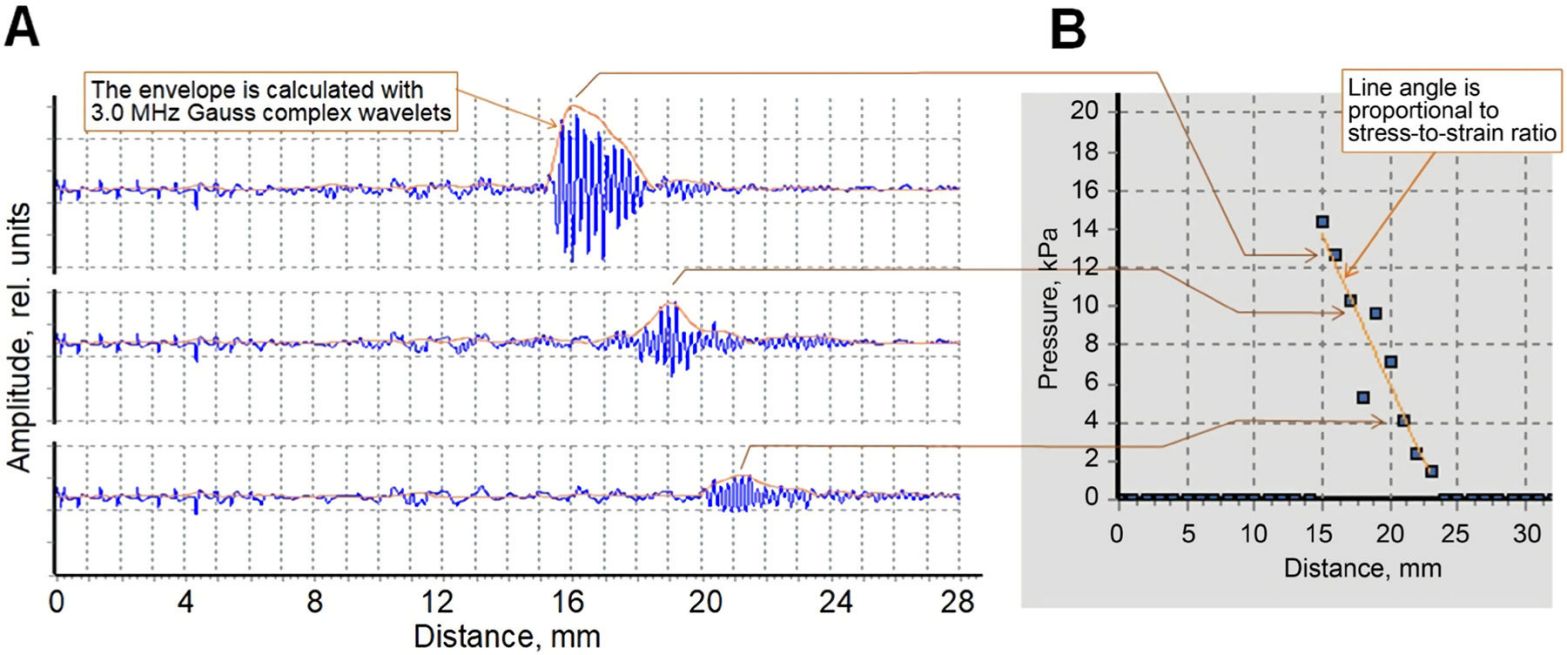
Measurement approach. (A) Ultrasound signals reflected from internal cervical os during cervix deformation by the probe; (B) Stress (pressure)-strain (compression) data recorded for 32 y.o. women at 25 week pregnancy.

Women at 22 – 29 weeks of pregnancy scheduled for a regular examination were considered eligible for the second part of the CM study enrollment. CM measurements were performed at an average gestational age of 25.4 ± 2.3 weeks (range, 22 – 29 weeks). The study of all ten women was successful. The recorded ultrasound signals had an identifiable peak amplitude reflected from the cervix’s internal os to allow reproducible measurement of ultrasound time-of-flight (see Figure 2). The peak position was calculated with the use of a signal envelope—see the light brown envelope line in the left panel of Figure 2.

**Figure 3.**
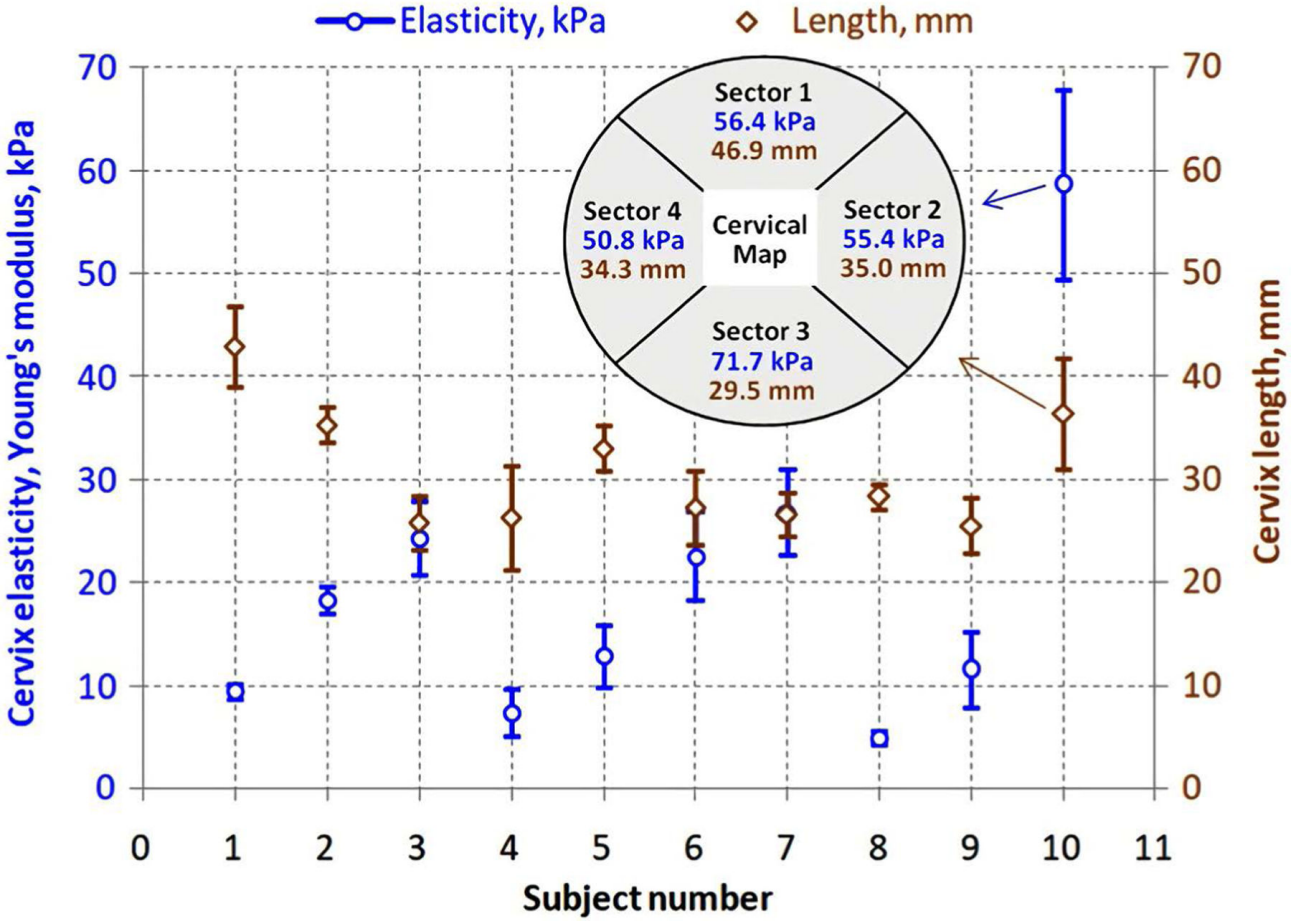
Cervical elasticity and length for 10 pregnant women measured by Cervix Monitor. Cervical Map with four sectors shows measurement results for subject number 10.

Figure 3 presents all CM data recorded for cases 1 – 10. The cervix map has four sectors; the results for one of ten cases (tissue elasticity and length distribution per four sectors) are shown in Figure 3. Average values and standard deviations (up/down bars) for cervical elasticity and length for 10 cases were calculated based on two measurements per 4 sectors (8 measurements per case); the values were 19.7 ± 15.4 kPa, and the length was 30.7 ± 6.6 mm. The average standard deviation for the 4 cervix sector measurements of elasticity was found as ±3.5 kPa and the length was ±3.4 mm.

**Figure 4.**
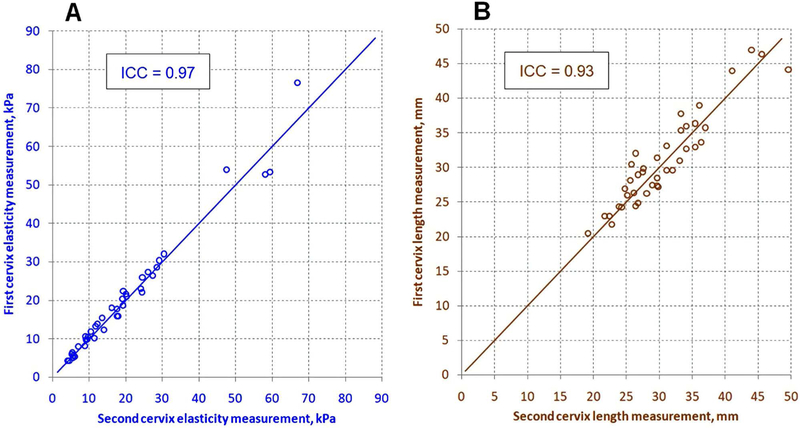
Relationship for two measurements. Intraclass correlation coefficients (ICC) for two measurements of cervical elasticity (A) and length (B) by the same operator.

**Figure 5.**
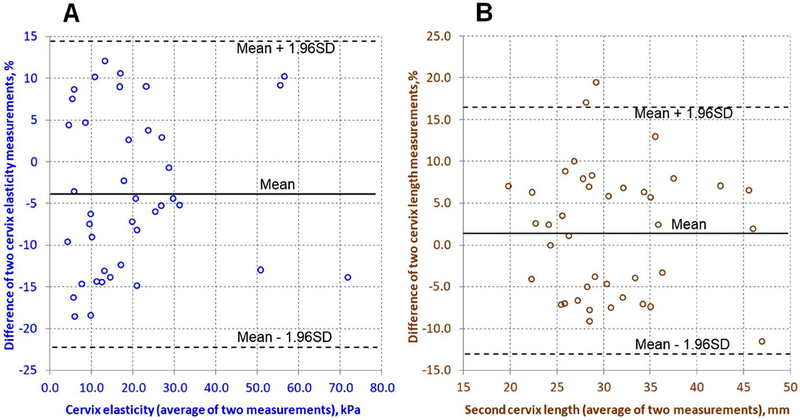
Scatter plots of difference between two measurements. Bland-Altman scatter plot of the percentage difference between two measurements of cervical elasticity (A) and length (B) by the same operator. The solid lines represent the proportionate mean difference; the dashed lines represent the 95% limits of agreement.

Measurement repeatability between two measurements at the same cervix sector on pregnant women was assessed with an intraclass correlation coefficient (ICC), as the correlation between any two measurements made on the same subject [34]. ICC for cervix elasticity was found to be 0.97 and for a cervical length of 0.93 (see Figure 4). The bias and the 95% limits of agreement of the cervical elasticity measurement are 3.8% and −22.4% to +14.9%, the precision was 9.5% (see left panel in Figure 5). The bias and the 95% limits of agreement of the cervical length measurements are −1.6% and −13.3% to +16.5%, the precision was 7.6% (see right panel in Figure 5).

The cervical length measurements with GE Voluson E8 (conventional ultrasound method) was not part of the protocol, but we obtained the measurements in 8 of 10 subjects that were studied. The Pearson correlation coefficient between cervical length measured with CM (average in sectors 1 and 3) and commercial ultrasound was found to be 0.48 for these 8 subjects; CM in average demonstrated 16.4% decrease versus conventional ultrasound in cervix length measurements.

The average level of pain reported by pregnant women was 1.7 on the scale from 1 to 4; the comfort level as 2.0 on a scale from 1 to 3. No adverse events with the CM were reported.

## 4. Discussion

The study has shown that the proposed tactile-ultrasound approach allows the measurement of cervical elasticity and length at an average gestational age of 25.4 ± 2.3 weeks with an acceptable precision of 9.5% and 7.6% consequently. It seems that the acceptable measurement reproducibility with the soft tissue elasticity measurements, being transformed into the Young’s module values, typically demonstrate a measurement accuracy of 3% - 15% and a measurement repeatability of 8% - 14% [31]. The cervical elasticity (average for 4 sectors) in this study ranged from 4.9 kPa to 58.6 kPa which constituted almost a 10-fold change from the lowest value. The 9.5% change (precision) from 32 kPa (average value in 4.9 kPa - 58.6 kPa range) amounts to 3.0 kPa, seems to be an acceptable proportion for elasticity measurement. The length of the cervix (average for 4 sectors) in this study ranged from 25.5 mm to 42.9 mm, which constitutes a 68% increase from the lower value. The critical changes in the cervical length are expected from 40 mm to 20 mm and 7.6% from the lower value will be 1.5 mm. The average standard deviation for the 4 cervix sector measurements seems to basically represent the variability by the cervix sectors which were found as ±3.5 kPa (elasticity) and ±3.4 mm (length). That means the reproducibility error of cervical length measurement of ±1.5 mm is capable to detect not only the length differences in pregnant women, but it allows resolution of the cervix anatomical variability by its 4 sectors. The +3.8% bias in the cervical elasticity measurement may be explained by the cervix strain hardening at the second measurement; the −1.6% bias in the cervical length measurement may cause cervix strain hysteresis at the second measurement (see Figure 5).

CM appeared to under measure the cervix length in comparison with the conventional ultrasound. This may be explained by cervix lengthen during the cervical measurement with commercial ultrasound because funneling may lessen. In contrary, measurement with CM may shorten the cervix because CM probe is targeted to compress cervix along its canal. It may be advantageous to measure with CM only through a portion of the cervix (whether it is anterior, posterior or lateral) because funneling may be removed, which can be dynamic, from the equation. It is important to note that absolute cervical length is not important for the cervical elasticity measurement.

The average level of pain reported by women was 1.4 on the scale from 1 to 4; the comfort level as 2.1 on a scale from 1 to 3. A speculum is generally considered more uncomfortable that a manual digital exam; it is expected that the examination with the CM using the speculum was more uncomfortable.

In the last decade, a new technology named elastography, or elasticity imaging, for measuring and the visualizing the soft tissue viscoelastic characteristics, has emerged. Two approaches for cervical ultrasound elastography for quantitative determination of the physical properties of the cervix, namely, strain elastography and shear wave elastography have been developed. We identified 11 clinical studies in the last three years, which tested the hypothesis that cervical elastography may be useful in predicting preterm delivery [20]-[25] [34] [35] [36] [37] [38]. Of the eleven studies identified, seven used strain ultrasound [20]-[25] [35] and four used shear wave ultrasound [34] [36] [37] [38]. These studies assessed between 30 and 628 subjects with a total of 1901 women in the eleven studies. The data from these works suggest that assessment of cervical elasticity may be a more useful predictor than simply measuring the length of the cervix. Predictive sensitivity and specificity were found to be in the range from 59.0% and 86.0% [25] to 96.7% and 87.0% for the two approaches, respectively [37]. In all these publications, the investigators noted that they felt that significant additional work was necessary before the measurement procedure can be standardized and made reliable.

Strain ultrasound elastography determines only the relative values of tissue elasticity because the applied transducer pressure is unknown. The shear wave ultrasound elastography provides, in principle at least, a more objective description of tissue elasticity; however, the cervical elasticity is described as a shear wave speed [34] [36] [37] [38], but not as Young’s modulus which requires solution of the inverse mechanical problem in absence of stress data. There are several difficulties in using this approach: 1) cervical tissue heterogeneity implies distortions in the shear wave elasticity estimates, 2) placing a transducer next to the cervix is likely to cause a tissue deformation, thereby causing a non-controllable increase in the tissue stiffening, 3) any movement should be avoided for 3 – 5 seconds with a shear wave transducer, and 4) it requires a special transducer [39]. Both these ultrasound techniques require premium level, expensive ultrasound equipment [40] [41]. A much simpler and a less expensive aspiration technique has significant limitations in the context of biomechanical characterization of the cervix: a) uncertainty of the applied force, and b) only a small volume of tissue, primarily on the distal cervix, is tested [11] [42]. The proposed approach with the CM allows a) direct acquisition of stress-strain data, and b) direct assessment of the cervix with Young’s modulus [30] [31].

The clinical risk factors for SPTD include obstetric history (*i.e*., familial genetic predisposition, uterine malformation, previous preterm labor, previous cervical surgery) and other aspects of the current pregnancy (*i.e*., multifetal gestation, genital tract bleeding and/or infection, fetal malformation, shortened cervix) [43] [44]. Current tests for the SPTD can be divided into three general categories: 1) risk factors, 2) cervical conditions, and 3) biochemical testing. Combining all of the risk factors still falls short of 50% in the prediction of pregnancies that deliver preterm [43] [45]. The biochemical markers (gestation tissues, biological fluid analysis, proteomic data) for the prediction of SPTD do not achieve the desired diagnostic accuracy [46] [47] [48] [49][50]. The cervical length, measured in the routine second-trimester transvaginal ultrasonography, is not sensitive enough to predict SPTD [19] [51] [52].

Extensive remodeling is needed for the cervix to dilate and pass a fetus completely. The extracellular matrix of the cervix is primarily made up of tightly packed collagen bundles. Gradually, throughout the pregnancy, the composition of the cervix changes as the collagen density decreases, and the realignment and degradation of collagen cross-linking due to proteolytic enzymes, and an increase in the hyaluronic acid and water content. Cervical softening and distention result from these extracellular matrix compositional changes [53].

This pilot study provided 1) the assessment of the proposed approach to measure cervix elasticity and length, 2) the highlights of its strong and weak aspects to be addressed in the probe and procedures modifications, and 3) the basis for extended prospective development and validation clinical studies.

The strength of this study lies in the novel approach for cervical elasticity and length. The cervical elasticity receives quantification in terms of Young’s modulus from stress-strain data. We acknowledge that our study has some limitations. First, one needs to make sure that the reflected ultrasound signal came from the cervix internal oz; it will be the subject of further research. Second, it seems that a cervix elasticity model must be incorporated into the cervix elasticity calculation which will take in account the strain distribution along measured cervix compression (strain distribution) by the probe. Third, the total studies sample size in the study is relatively small. A study with a larger number of cases would enable us to explore the entire range of the cervical conditions and focus more on the prediction of SPTD at the gestational age when clinical procedures could prevent the SPTD. Yet, this is the first study using CM for this purpose, and the current study will serve as the basis to guide the design of future protocols.

The novelty of this work is the implementation of the tactile and ultrasound transducers in one cervical probe and demonstration of feasibility of proposed approach for measurement of cervical elasticity and length on pregnant women.

## 5. Conclusion

This study has demonstrated clinically acceptable measurement performance and reproducibility based on the acquisition of stress-strain data by tactile and ultrasound transducers. Availability of the stress-strain data allowed the computation of cervical elasticity and length. This approach has the potential to provide cervical markers in the prediction of spontaneous preterm delivery. Further research is needed.
